# Implementing a Positive Learning Experience Related to Older Adults for Undergraduates: A Toolkit

**DOI:** 10.1093/geroni/igaa057.1781

**Published:** 2020-12-16

**Authors:** Ann Scheve, Elizabeth Bruderle

**Affiliations:** Villanova University, Villanova, Pennsylvania, United States

## Abstract

Undergraduate nursing students are frequently exposed to older adults in the clinical setting, where they assess and manage their diseases and its consequences. But that is not enough! To support healthy aging, students need positive intergenerational learning experiences with older adults to discover the gifts of aging early in their curriculum. The goal of these experiences is to help students reflect on their thoughts about aging and reframe how they view older adults. During this presentation we will provide a tool kit based on our experience incorporating positive intergenerational learning early in our curriculum, offer practical guidelines and share constructive feedback.

